# Isolation and characterization of phytoconstituents from *Chlorophytum borivilianum*

**DOI:** 10.4103/0974-8490.75452

**Published:** 2010

**Authors:** Sharada L. Deore, Somshekhar S. Khadabadi

**Affiliations:** *Government College of Pharmacy, Kathora Naka, Amravati - 444 604, MS, India*

**Keywords:** *Chlorophytum borivilianum*, Chlorophytoside-I, fatty acids, stigmasterol

## Abstract

**Background:**

The present communication deals with the identification and characterization of bioactive principles from the roots of Chlorophytum borivilianum.

**Method:**

Methanolic extract and its fractions were used to isolate different phytoconstituents. The structures of isolated compounds were characterized and elucidated with chemical and spectroscopic techniques such as Infra Red, Nuclear Mass Resonace and Mass spectroscopy experiments. Fatty acids were characterized by GC-MS analysis.

**Result:**

Three Fatty acids were isolated and confirmed. One sterol stigmasterol was isolated. One new saponin named as Chlorophytoside-I (3β, 5α, 22R, 25R)-26-(β-D-glucopyranosyloxy)-22-hydroxy-furostan-12-one-3 yl O-β-D-galactopyranosyl (1-4) glucopyranoside was isolated.

**Conclusion:**

The roots of Chlorophytum borivilianum contain three important fatty acids, common sterol stigmasterol and one furostanol saponins.

## INTRODUCTION

*Chlorophytum borivilianum* belonging to family Liliaceae is a plant well-known for its aphrodisiac as well as adaptogenic activity in India. Roots are claimed to be useful to treat oligospermia, pre- and post-natal symptoms, arthritis, diabetes and dysuria. Saponin, polysaccharides and mucilage are the major constituents of *Chlorophytum borivilianum*. Recently, four new steroidal saponins have been isolated from plant tuber. The present constituents have not been reported previously.

## MATERIALS AND METHODS

### Instrumentation

Open-column chromatography silica gel 60 as stationary phase, n-hexane, chloroform and methanol by gradient elution technique for sterols.

Chloroform - methanol as a gradient mobile phase for saponin isolation

### TLC Silica gel F254 (Merck)

HPTLC Silica gel F254 (Merck); Camag HPTLC automatic sample applicator (Linomat 5), Scanner 3 and UV cabinet

### Solvent systems

For fatty acids Petroleum ether Diethyl Ether (4.2:2) as a mobile phase and sprayed with 5% Methanolic Sulphuric acid

For sterol Toluene ethyl acetate, 82, sprayed with Anisaldehyde Sulphuric acid

For saponins chloroform:Glacial acetic acid: MeOH: Water, 16:8:3:2) sprayed with Anisaldehyde -sulphuric acid reagent

### HPLC Jasco binary system

For sterol Acetonitrile/methanol (50/50, v/v) containing 3% water (v/v) was used as a mobile phase and C18 as a column. The flow rate was 1 ml/min

### For saponin

GC-MS (IIT, POWAI, Mumbai) Hewlett Packard GCD-1800 A EI Source, Quadrupole Analyzer, Mass range 10-425 amu

Optical rotation AA-OR automatic polarimeter.

UV-spectra Shimadzu 1700

FTIR Spectra Nicolet Instruments Corporation, USA MAGNA 550, Range - 4000 cm-1 to 50 cm-1 and Shimadzu IRAffinity-1 (4000\-350 cm-1) Kbr

1D- and 2D-NMR Spectra (CDRI, Lucknow) Bruker DRX-300 (300MHz FT NMR)

Fast-atom-bombardment [FAB) MS neg., CDRI, Lucknow) Mass Spectrometers Joel SX 102 (EI/CI/ FAB)

### Extraction Methodology

The tubers (12 kg) were chopped up into small pieces and defatted with pet. ether for 6 h. Filter and filtrate was evaporated to yield yellow semisolid mass. Then the dried marc was soaked in MeOH for seven days. This methanolic extract was concentrated under reduced pressure to obtain dark-colored residue (25 g). This residue was dissolved in water and then partitioned with n-butanol. n-butanol layer was separated and distilled off to yield crude saponin. This residue was used for the isolation of saponin compounds by column chromatography. TLC of methanol, butanol fractions was developed in suitable solvent system [CHCl3: Glacial acetic acid: MeOH: Water, 16:8:3:2 derivitised with Anisaldehyde -sulphuric acid reagent] was given five separated spots.

### Fatty acid isolation[1]

A part of petroleum ether extract was dissolved in pet. ether solvent and subjected to TLC studies by using silica gel G as stationary phase, Petroleum ether Diethyl Ether (4.22) as a mobile phase and detection was done by 5% Methanolic Sulphuric acid. TLC showed three well-separated spots. Co-TLC partially confirmed that extract contains linoleic acid, pthalic acid. For isolation of fatty acids the extract was subjected to saponification. The free fatty acid further derivitised to methyl ester which is most preferred form of fatty acids for GC-MS analysis. Petroleum ether extract was evaporated under vacuum to obtain fatty oil. The oil (0.2 to 0.4 mg) was hydrolyzed with 0.5 N potassium hydroxide in ethanol (15 ml) for 4–6 h. After Hydrolysis free fatty acids were separated. To obtain unsaponified fraction of this free fatty acid it was then extracted with diethyl ether (10 ml) and allowed to stand for a few minutes. Upper layer was collected to get unsaponified part and lower water layer to get free fatty acids. Hydrochloric acid water was added to make the water layer (containing the free fatty acids) acidic and extracted with diethyl ether for complete separation of free fatty acids. This ether layer further completely evaporated at room temperature. This free fatty acid further derivitized to methyl ester by addition of 25 ml of 2% methanolic sulphuric acid with 6 hr heating in water bath. Ether and 1% aqueous potassium hydroxide was added and ether layer was separated. The aqueous layer was extracted twice again with ether. Finally, combined ethyl layers were washed with distilled water. The ether layer was dried over anhydrous sodium sulphate and finally transferred to Ependroff’s tubes for GC-MS analysis.

### GC-MS analysis

The methyl esters of fatty acids were analyzed using a Shimadzu QP 5050 equipped with reference libraries using SE-52 (Mega, Legnano, Italy) cross-linked fused-silica capillary column coated with 5% phenyl-polymethylsiloxane (25m*0.25mm i.d.*0.25um film thickness); column temperature, 60° C (8 min). Helium was used as carrier gas, using 122.2KPa (51.6 cm/sec).

### Sterol Isolation[2–5]

Part of Petroleum ether extracts was utilized to isolate sterols. Petroleum ether extract was partitioned with CHCl3 subjected to column chromatography using silica gel (Mesh 60-120; wet packing method). The column was run with n-hexane, chloroform and methanol by gradient elution technique. TLC was used to monitor the elutes. Similar fractions were pooled together to yield CT (200 mg) which was further again purified by preparative TLC. This compound was identified as Stigmasterol (30 mg) by comparing TLC, HPLC, IR and 1H NMR data with the literature.

### Saponin Isolation[6–9]

The nBuOH fraction (3.2 g) was subjected to column chromatography using Silica gel and CHCl3 MeOH as a gradient mobile phase. Fractions were monitored by TLC and similar fractions combined to give mixture of two saponins S1 and S2. Both saponins were further separated by preparative TLC. Quantity of S3 was not found enough to do structural elucidation.

Acid hydrolysis: To identify the sugar moieties saponin S1 (further coded as CS) was hydrolyzed with HCL to separate sapogenins and sugar. A solution of saponin S1 (2 mg) in water (2 ml) was treated with 2N aq. HCL (5 ml) and refluxed for 3 h. After extraction with CH2Cl2 (5 ml), the aq. layer was repeatedly concentrated with MeOH until neutral, and then analyzed by TLC (silica, CHCl3/MeOH/H2O, 8-5-1) with authentic samples galactose (Rf 0.21), glucose (Rf 0.23).

Saponin S1 (CS) (3*β*, 5*a*, 22*R*, 25*R*)-26-(*β* -D-glucopyranosyloxy)-22-hydroxy-furostan-12-one-3 yl *O-β*-D-galactopyranosyl (1-4) glucopyranoside.

## RESULTS AND DISCUSSION

**Figure 1:**
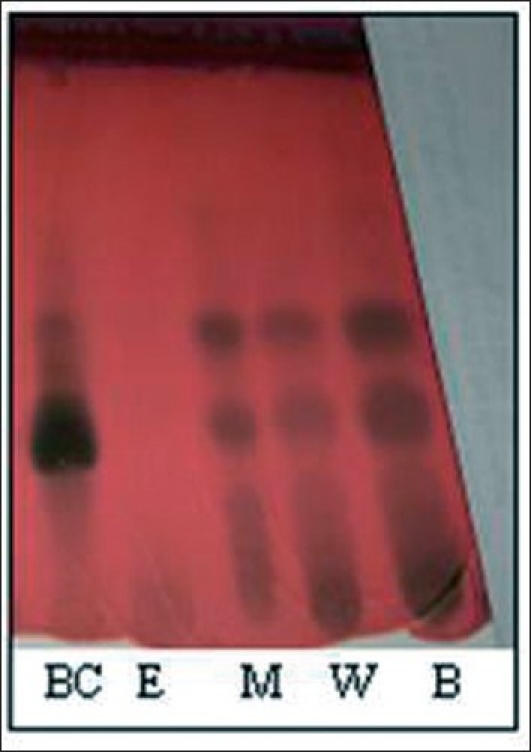
TLC of separated fractions. (BC: Butanol Fraction of Column Chromatography, E: Ethyl acetate fraction, B: Butanol Extract, W: Water Extract, M: Methanol Extract)

Petroleum ether and methanolic extract was prepared and used for isolation of active constituents. TLC profile [Figure 1] of extracts was prepared to get exact idea about the constituents present in different extracts.

### Fatty Acids

Fatty acids are carboxylic acids with long hydrocarbon chains. The hydrocarbon chain length may vary from 10-30 carbons (most usual is 12-18). Fatty acids also called as aliphatic acids are an important source of energy stored in the form of triglycerides and act as intermediates in the biosynthesis of polyketides and hormones. Commercially, fatty acids and their derivatives are useful in the manufacturing of food, cosmetics and toiletries products, soaps, papers, plastic, varnishes, paints and insecticides. Fatty acids can be saturated and unsaturated, depending on double bonds.

Essential fatty acids belong to the class of PUFAs. There are two types of essential fatty acids, omega-6 and omega-3 fatty acids which can be short-chain (omega-3α: Linolenic acid, omega-6: eicosapentaenoic acid or EPA, docosahexaenoic acid DHA) or long-chain polyunsaturated fatty acids (omega-3α Linoleic acid, omega-6: gamma-linoleic acid or GLA, dihomo-gamma-linolenic acid or DGLA, arachidonic acid or AA). Linoleic acid and isomers of linolenic acid are EFA as its derivative linolenate blocks synthesis of prostaglandins and thus can be useful in the treatment of various ailments, especially cardiovascular disease. Linoleic acid has been found to be an important compound in the prevention of hair loss, cancer, cystic fibrosis and dermatitis.

**Figure 2:**
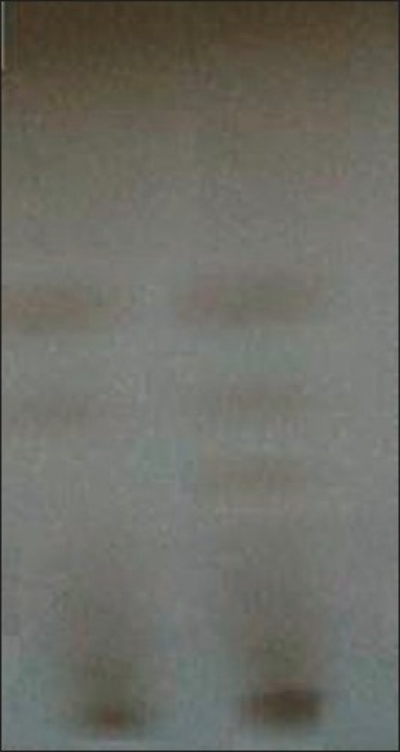
TLC of fatty acids. Where PE Petroleum ether extract, L Linoleic acid

**Figure 3:**
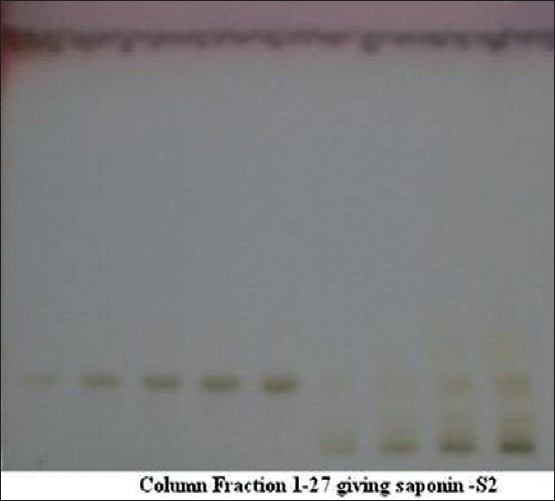
TLC of isolated saponin S2

**Figure 4:**
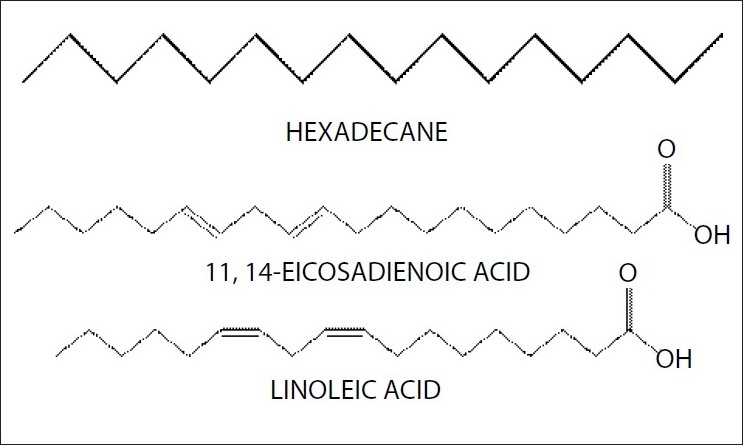
Structure of isolated fatty acids

**Figure 5:**
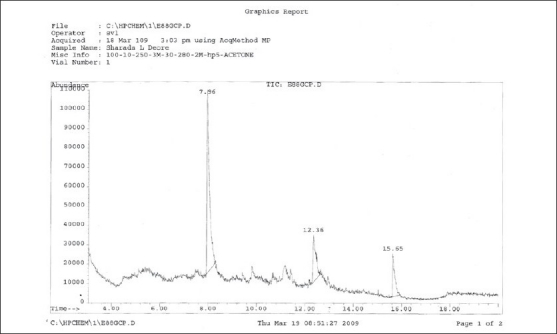
GC-MS analysis of fatty acid mixture (SAIF, IIT, Mumbai)

Part of pet. ether extract was used to separate fatty acids. Co-TLC studies [Figure 2] have also confirmed the presence of linoleic acid but 11, and 14-Eicosadienoic acid and hexadecane has been confirmed after GC-MS analysis at retention time 7.96 (linoleic acid), 15.65 (11, 14-Eicosadienoic acid) and 12.36 (hexadecane). GC-MS analysis [Figures 3 and 4] of methyl ester derivatives of the pet. ether extract has shown presence of linoleic acid, hexadecane and 11, and 14-Eicosadienoic acid [Figure 5].

Thus the use of tubers of CB in inflammation, arthritis and cardiovascular diseases may be due to presence of these isolated fatty acids.

### Sterol

The compound CT is obtained as amorphous powder and gave a positive Liebermann-Burchard test for a steroid with MP 162-164°C. UVmax: 257 nm; IR (KBr) cm-1: 3394.3cm-1(O-Hstr.); 3218cm-1 (-HC==CH cyclic); 2940.8cm-1(C-Hstr.); 2868.4-1cm-1(C-Hstr.); 1617.5cm-1(C=C absorption peak);1H-NMR (CDCl3, 400 MHz): δ 3.52(m), 5.358(br, s), 0.68(s), 1.01 (s), 0.92(d, J= 6.4), 0.814 (d, J=6.5), 0.833 (d, bj=6.5) and 0.845 (d, j=7.5) ppm. Other peaks were observed at δ 3.52(m), 5.357 (br, 5), 0.699(s), 1.02(d, j=7.5), 0.795(d, j=6.5),0.846 (d, j=6.5) and 0.804 (t, j=7.5) ppm.

**Figure 6:**
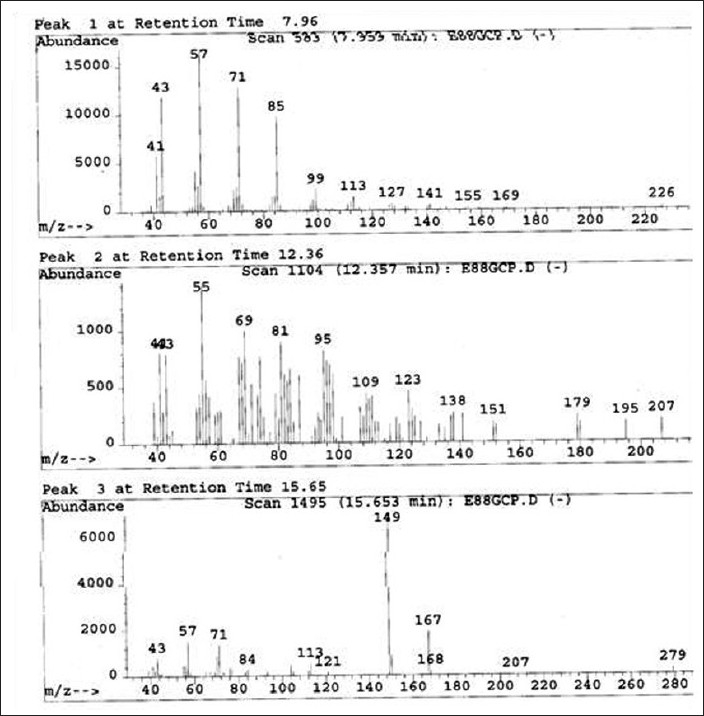
GC-MS analysis of fatty acid mixture (SAIF, IIT, Mumbai)

**Figure 7:**
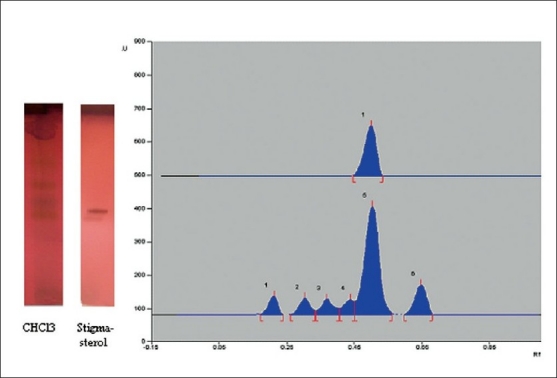
HPTLC of standard stigmasterol and chloroform fraction

**Figure 8:**
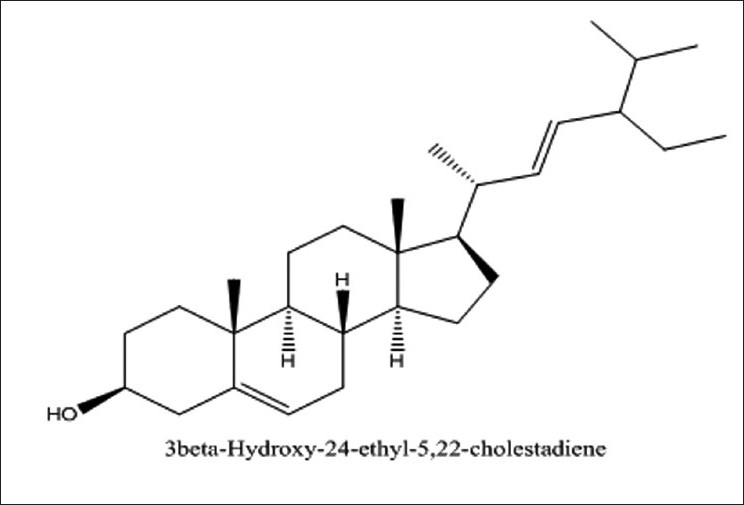
Structure of isolated stigmasterol (C29H48O)

**Figure 9:**
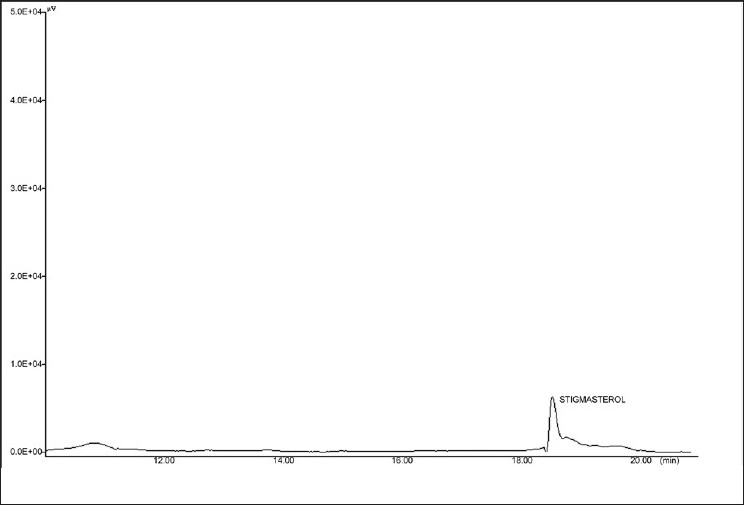
HPLC of isolated sterol

HPTLC and HPLC studies of sterol are shown in Figures 6 and 7. The assignments shown by compound sterol for IR and HNMR spectroscopy are found to be matching with stigmasterol [Figure 8]. The IR values [Figure 9] at 3320 cm-1, 1220 and 680 cm-1 indicate the O-H bond vibrations of the hydroxyl group. C-H vibrations of the unsaturated part were observed at 890 cm-1. C-C vibrations were found at 1648 cm-1. The stretching and bending vibrations of methyl part were noticed by the intense band 2946 cm-1 and medium intensity band at 1450 cm-1.

**Figure 10:**
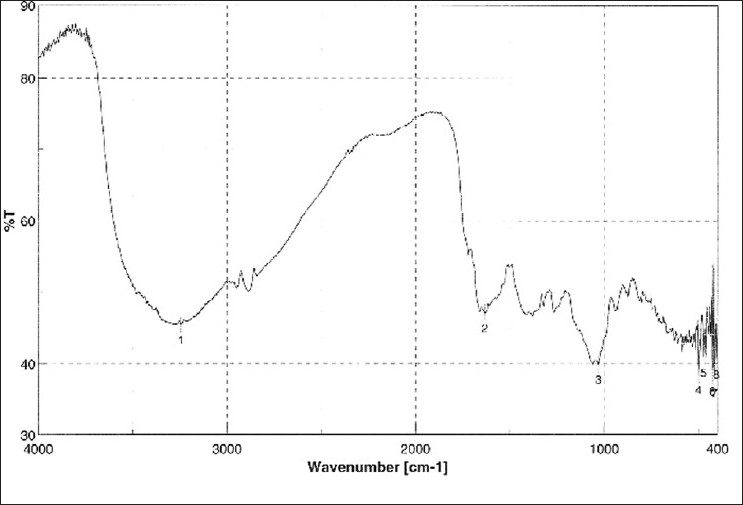
IR spectra of isolated sterol

In lH-NMR spectrum [Figure 10] of CT, H-3 proton at S 3.62 (J 4.5, 1.1 MHZ) and H-6 olefinic proton at S 5.14. Two olefenic protons of H-22 and H-23 appeared downfield at S 4.16 (m) and S 4.14 (m) respectively. Six CH3 protons were found at S 1.27, S 1.19, S 1.07, S 1.00, S 0.99 and S 0.91 (3H each, s, CH3).

**Figure 11:**
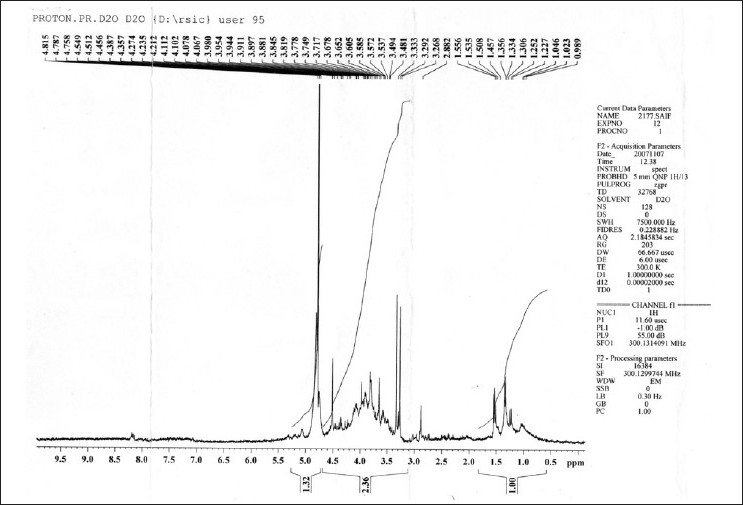
HMNR spectra of isolated sterol (SAIF, CDRI, Lucknow)

The FAB spectrum [Figure 11] showed the peaks at 412, 394, 255, 213, 199, 159, 133, 121, 105, 91, 87, 69 which are in good agreement with reported values of the structure of stigmasterol.

The identification of component was based on direct comparison of the TLC (Toluene: ethyl acetate, 8:2 sprayed with Anisaldehyde Sulphuric acid) NMR spectral data with those for standard compounds, computer Library matching, as well as comparison with HPLC study of isolated sterol (Acetonitrile/methanol (50/50, v/v) containing 3% water (v/v) was used as A mobile phase and C18 as a column). The flow rate was 1 ml/min. The RT of stigmasterol was found to be 18.3 min.

### Saponin

**Figure 12:**
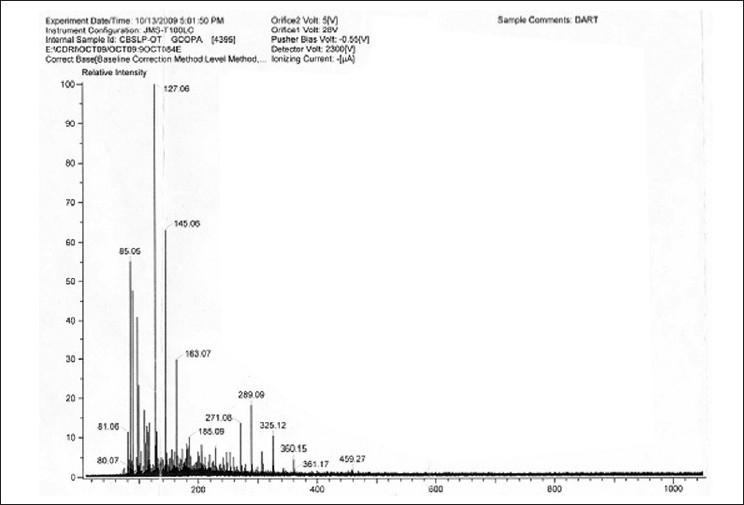
Mass spectra of isolated sterol (SAIF, CDRI, Lucknow)

**Figure 13:**
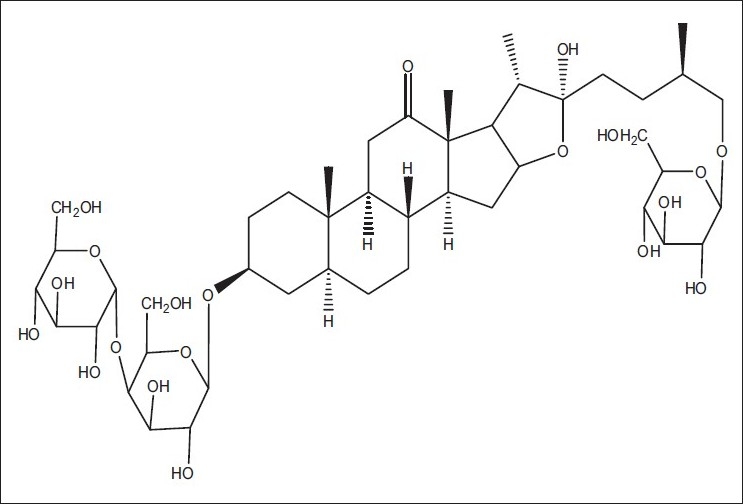
structure of chlorophytoside- I

**Figure 14:**
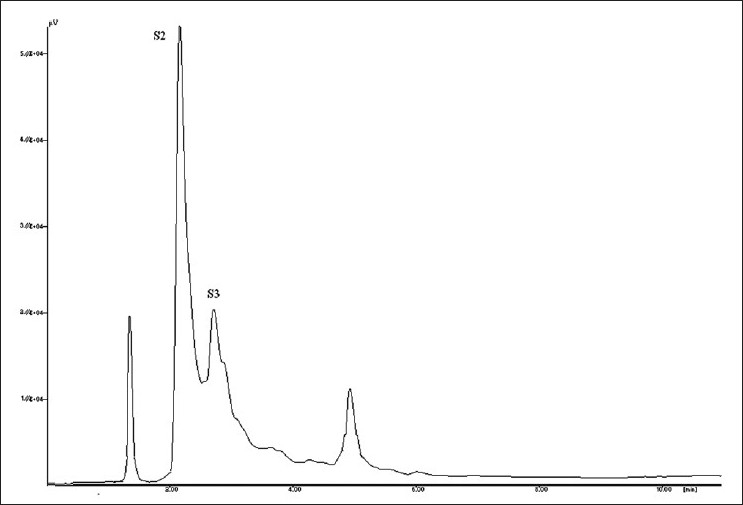
HPLC chromatogram of isolated saponin

**Figure 15:**
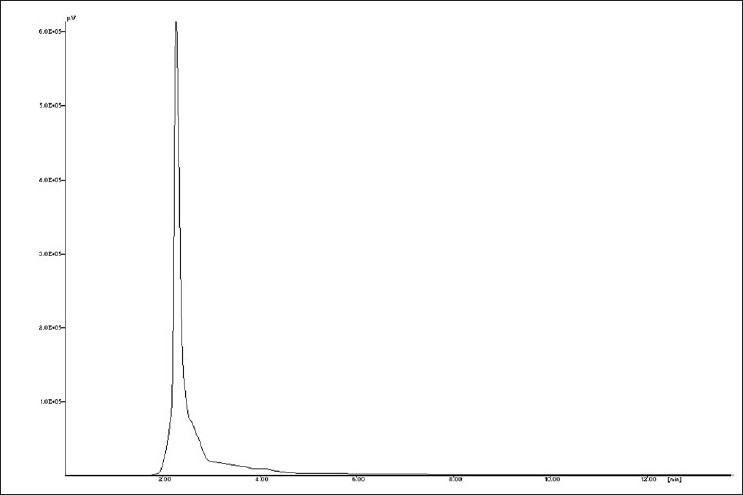
HPLC chromatogram of saponin content of methanolic extract

Saponin S1 [Figure 12] was obtained as white amorphous powder and showed characteristic colors typical for furostanol saponins in both Ehrlich reagent test and Liebermann-Burchard test, suggesting that S1 is a furostanol saponin with RF value 0.23 in Chloroform: Glacial acetic acid: methanol: water [64:32:12:8] [Figure 13]. HPLC studies of ME extract by C18 with acetonitrile: water (3:7) at 1ml/min flow rate showed presence of four saponins [Figures 14 and 15]. The HPLC of isolated saponin was performed by same method which has given single peak at Retention Time of 2 min. This peak was also present in HPLC graph of methanolic extract.

**Figure 16:**
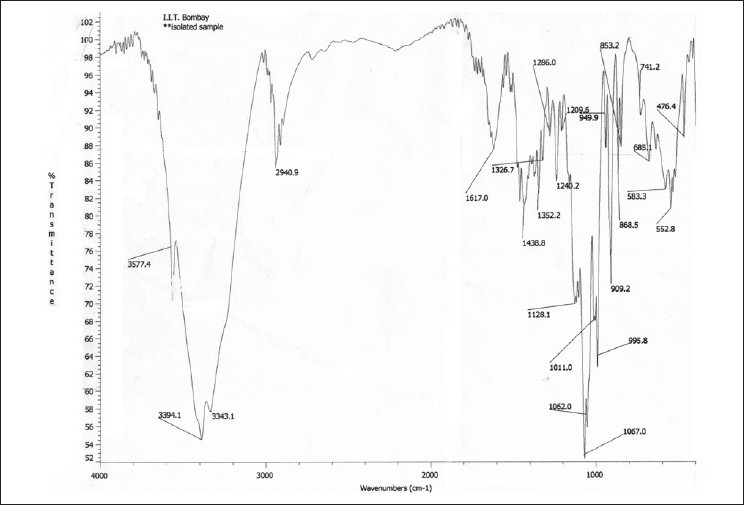
IR spectra of isolated Saponin CS

Molecular formula- C45H74O20 molecular weight- 935.06; [α] D25 -63.6° (c 0.25; MeOH); MP 212-214°C. UVmax: 278 nm; IR^KBr^ V_max_ cm-1: 3400-3350 (OH), 2364, 2085, 1780, 1757, 1657, 1584, 1112, 617. H-NMR and 13C-NMR values are given in Table 1 and 2. FAB m/z: 937.64 [M+H]+, 775(M+H-162)+, 629 (M+H-162-146)+, 465 (M+H-162-146-164)+, 303 (M+H-162-146-164-162)+ and few additional peaks like 450 (M+H-162-326)+, 432 (M+H-162-326-H20)+, 303 (M+H-162-326-146). The IR spectrum [Figure 16] of S1 showing peaks at 3350-3400 and 1600-1700 are characteristic furostanol saponins.

**Table 1:** ^13^C NMR and 1H- NMR values for saponin Chlorophytoside –I

Sr. No.	13C NMR	1H- NMR	Sr. No.	13C NMR	1H- NMR
	Delta values in ppm			Delta values in ppm	
CH2-1	41.5	1.57, 1.31	CH2-15	32.5	1.74, 1.48
CH2-2	32.4	1.71, 1.46	C-H-16	81.5	3.73
C-H-3	70.95	3.2	C-H-17	69.09	2.03
CH2-4	38	1.64, 1.33	CH3-18	19	1.31
C-H-5	45	1.42	CH3-19	20.4	1.03
CH2-6	28	1.57, 1.31	C-20	44.3	2.37
CH2-7	28.2	1.56, 1.28	CH3-21	16.2	0.96
C-H-8	36	1.41	C-22	103	-
C-H-9	61	2.02	CH2-23	35	1.57
C-10	60.2	-	CH2-24	21	1.25
CH2-11	41	2.32, 2.03	C-H-25	39	1.98
CH2-12	212	-	CH2-26	69.57	3.45, 3.21
C-13	55	-	CH3-27	18	0.89
C-H-14	62.28	1.71	C22-OH		3.65

**Table 2:** ^13^C NMR and 1H- NMR values for sugars attached to Chlorophytoside –I.

Sr. No.	13C NMR	1H- NMR
	Delta values in ppm	
C-3 Gal		
1	109.8	5.03
2	74.4	3.73, 3.58
3	74.1	3.73, 3.58
4	83.1	3.02
5	79.6	4.00
6	62.5	3.79, 3.54, 3.65
C-3 Glu		
1	110.4	5.03
2	74.1	3.73, 3.58
3	76.8	3.49, 3.58
4	71.5	3.40, 3.58
5	81.5	3.76
6	62.2	3.79, 3.54, 3.65
C-26 Glu		
1	112.3	5.03
2	73.8	3.73, 3.58
3	76.8	3.49, 3.58
4	71.5	3.40, 3.58
5	81.5	3.76
6	62.2	3.79, 3.54, 3.65

**Figure 17:**
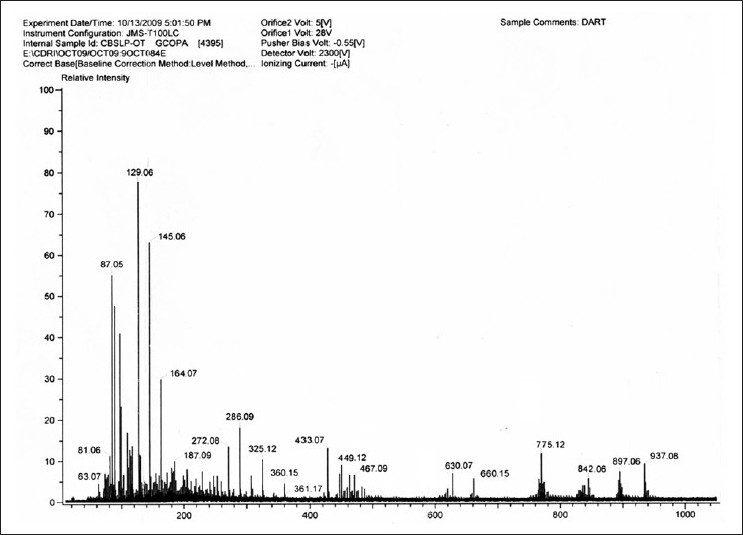
FAB spectra of isolated Saponin CS (SAIF, CDRI, Lucknow)

The FAB fragmentation pattern [Figure 17] confirms the removal of glucose moiety (MW 162) followed by side chain (MW 146) and the galactoglucopyranoside moiety (MW 326) along with few more common fragments peaks like that of basic steroid moiety at 255, 162, 326, 144 etc.

**Figure 18:**
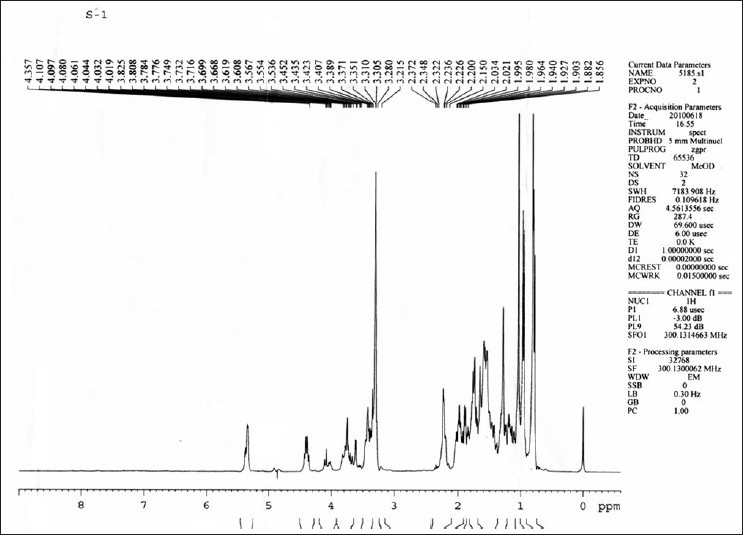
H-NMR spectra of isolated Saponin CS (SAIF, CDRI, Lucknow)

1H-NMR spectrum [Figure 18] of saponin S1 showed diagnostic signals of three methyl groups at δ 0.86 (3H, s, H3-18), 0.89 (3, s, H3-19), 1.62 (3H, s, H3-21), 0.90 (3H, d, J = 6.8 Hz, H3-27), and signals of two oxymethines at δ 3.81 (1H, m, H-3), 4.43 (1H, m, H-16) and one oxymethylene at δ 3.48 (1H, dd, J = 7.5, 9.5 Hz, Ha-26), 4.07 (1H, m, Hb-26), and four anomeric proton doublets at δ 4.79 (1H, d, J = 7.3 Hz, gal H-1), 5.06 (1H, d, J = 7.6 Hz, glc H-1), 4.70 (1H, d, J =7.6 Hz, glc'-H-1').

**Figure 19:**
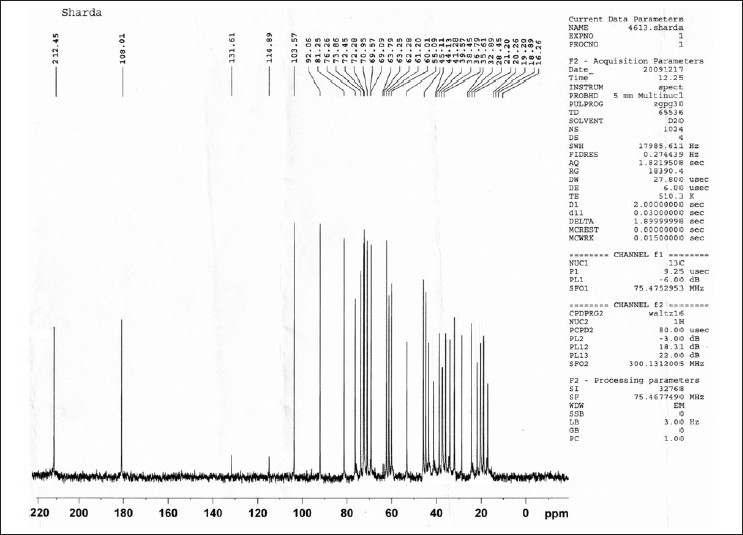
^13^C-NMR spectra of isolated Saponin CS (SAIF, CDRI, Lucknow)

A 13C-NMR spectrum is [Figure 19] also showed characteristic peaks for glucose, galactose and furostanol type saponin. From the physical, chemical and spectral evidences of the compounds it is concluded that the tubers of *C. borivilianum* contain stigmasterol and one new furostanol saponin named as (3*β*, 5*a*, 22*R*, 25*R*)-26-(*β* -D-glucopyranosyloxy)-22-hydroxy-furostan-12-one-3 yl *O-β*-D-galactopyranosyl (1-4) glucopyranoside.
